# Association between complete blood cell count-derived inflammatory biomarkers and gallstones prevalence in American adults under 60 years of age

**DOI:** 10.3389/fimmu.2024.1497068

**Published:** 2025-01-10

**Authors:** Chang Fu, Junhong Chen, Yongxin Wang, Yibo Yang, Xiaocong Li, Kai Liu

**Affiliations:** ^1^ Department of Hepatobiliary and Pancreatic Surgery, General Surgery Center, The First Hospital of Jilin University, Changchun, China; ^2^ Department of Pharmacy, China-Japan Friendship Hospital, Beijing, China; ^3^ Clinical Trial Research Center, China-Japan Friendship Hospital, Beijing, China

**Keywords:** CBC-derived inflammatory indicators, gallstones, NHANES, cholelithiasis, inflammation

## Abstract

**Background:**

The trend of gallstones occurring in younger populations has become a noteworthy public health issue. This study aims to investigate the association between complete blood cell count (CBC)-derived inflammatory indicators and gallstones in adults under 60 years of age in the United States.

**Methods:**

This cross-sectional study used data from the National Health and Nutrition Examination Survey (NHANES) from 2017 to 2020. Associations between CBC-derived inflammatory biomarkers and gallstones were assessed using multivariable logistic regression models, with results presented as odds ratio (OR) and 95% confidence interval (CI). Restricted cubic splines (RCS) were employed to examine potential non-linear relationships. Subgroup analyses were also conducted to explore differences across population subgroups.

**Results:**

This study comprised 4,977 participants, among whom 398 were diagnosed with gallstones. After adjusting for confounding variables, the highest quartile of systemic inflammation response index (SIRI) [OR (95%CI): 1.65(1.12,2.43)], systemic immune-inflammation index (SII) [OR (95%CI): 1.53(1.05,2.25)], monocyte-to-lymphocyte ratio (MLR) [OR (95%CI): 1.66(1.16,2.37)], and pan immune inflammatory value (PIV) [OR (95%CI): 1.82(1.23,2.71)] were associated with a significantly increased risk of gallstones compared to the lowest quartiles. RCS plots indicated a nonlinear relationship between several inflammatory biomarkers and gallstones.

**Conclusion:**

Our study found that SIRI, SII, MLR, and PIV can serve as clinical indicators for predicting the risk of gallstones in adults under 60 years of age in the United States.

## 1 Introduction

Gallstones are one of the most common digestive system diseases, with a high global prevalence of approximately 6%, imposing a substantial burden on healthcare systems and society (1). Gallstones form through the deposition of cholesterol or bilirubin in bile, with over 90% primarily composed of cholesterol (2). The risk factors for gallstones include both genetic and environmental factors, such as race, female, age, metabolic syndrome and dietary habits (3, 4). While many gallstones are asymptomatic, they may progress to severe conditions such as acute cholecystitis, cholangitis, and pancreatitis, significantly impacting quality of life (5, 6). Moreover, gallstones are also a high-risk factor for gallbladder cancer and cholangiocarcinoma, both of which have poor prognosis (7, 8). Currently, no widely accepted indicators exist for predicting the risk of gallstone formation. Therefore, identifying reliable and practical clinical indicators to assess gallstone risk is essential for effective prevention and timely intervention.

Inflammation plays a crucial role in gallstone formation, and conditions such as obesity, diabetes, and other inflammation-related metabolic disorders are strongly associated with gallstone development (9–11). Inflammatory proteins are correlated with circulating inflammatory markers in bile, and inflammasome activation also contributes to gallstone formation (12, 13). Complete blood cell count (CBC)-derived inflammatory indicators, including the systemic inflammation response index (SIRI), systemic immune-inflammation index (SII), neutrophil-to-lymphocyte ratio (NLR), monocyte-to-lymphocyte ratio (MLR), platelet-to-lymphocyte ratio (PLR), and pan immune inflammatory value (PIV), effectively reflect the body’s inflammatory status. These markers integrate monocyte, neutrophil, platelet, and lymphocyte counts, providing a comprehensive assessment of the immune-inflammatory state compared to single inflammatory indicators (14). Previous studies have validated the predictive value of CBC-derived inflammatory biomarkers in cardiovascular diseases, non-alcoholic fatty liver disease (NAFLD), sarcopenia, and asthma (15–18). However, the relationship between CBC-derived inflammatory biomarkers and gallstone disease remains unclear.

We utilized a representative sample from the National Health and Nutrition Examination Survey (NHANES) to investigate the potential association between CBC-derived inflammatory biomarkers and gallstone disease. This study aims to provide new insights into interpreting gallstone risk from an immune-inflammatory perspective.

## 2 Methods

### 2.1 Study design

NHANES is a nationwide survey conducted by the National Center for Health Statistics (NCHS), comprehensively collects data on demographics, physical measurements, medical history, nutritional intake, and health behaviors. The protocol of NHANES has been approved by the Ethics Review Board of the National Center for Health Statistics, and data are updated every two years.

**Figure 1:**
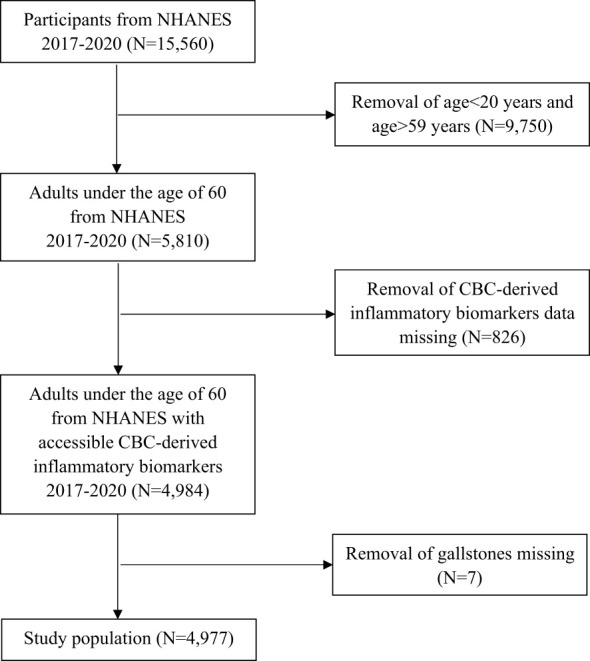
Flowchart for study population.

For this study, we used data from 2017 to 2020, as gallstone-related questionnaire responses were available only during this period. Over this timeframe, a total of 15,560 individuals participated in the survey. We sequentially excluded 9,750 participants who were either under 20 or over 59 years old, 826 participants lacking inflammatory biomarker data (neutrophils, lymphocytes, monocytes, platelet counts, and high-sensitivity C-reactive protein), and 7 participants missing gallstones data (**Figure 1**). The final study population consisted of 4,977 individuals.

### 2.2 Study variables

In this study, the exposure variables were CBC-derived inflammatory biomarkers, and the outcome variable was the presence of gallstones. CBC were measured using automated hematology analyzing devices (Coulter^®^DxH 800 analyzer) and reported as ×10^3^ cells/μL. CBC-derived inflammatory biomarkers were calculated using the following formulas: SIRI = neutrophil counts × monocyte counts/lymphocyte counts, SII = platelet counts × neutrophil counts/lymphocyte counts, NLR = neutrophil counts/lymphocyte counts, PLR = platelet counts/lymphocyte counts, MLR = monocyte counts/lymphocyte counts, PIV = SII×monocyte counts. The diagnosis of gallstones was based on participants’ responses to the question: “Has a doctor or other health professional ever told you that you have gallstones?”

Covariates included age, gender, race, body mass index (BMI), education level (categorized as less than high school level, high school, and more than high school level), marital status (married or with a partner unmarried), family poverty-to-income ratio (PIR), history of diabetes and hypertension (based on the questionnaire, participants who answered yes were identified as having these diseases), alcohol consumption (defined by affirmative responses to the question “Had at least 12 alcohol drinks/1 yr?” on the questionnaire ALQ101) and smoking status (defined by affirmative responses to the question “Smoked at least 100 cigarettes in life?” on the questionnaire SMQ020).

### 2.3 Statistical analysis

Continuous variables were described as mean ± standard deviation (SD) and categorical variables were described as counts (percentages). Differences across quartile groups of CBC-derived inflammatory biomarkers were assessed using the chi-square test, Student’s t-test or the Kruskal-Wallis test, as appropriate. The association between CBC-derived inflammatory biomarkers and gallstones prevalence was assessed using logistic analysis. The CBC-derived inflammatory biomarkers were categorized into quartiles and analyzed as categorical variables. The multivariate analysis was conducted using three models: Model 1 was unadjusted; Model 2 was adjusted for gender, age, and race; Model 3 included additional adjustments for education level, marital status, PIR, BMI, alcohol consumption, smoke status, hypertension and diabetes. To explore potential non-linear associations, restricted cubic splines (RCS) with four knots placed at the 20th, 40th, 60th, and 80th centiles were employed. Stratified analyses were used to examine the consistency of associations across subgroups. Two-tailed statistical tests with P < 0.05 were considered statistically significant and all statistical analyses were conducted using SAS 9.4.

## 3 Results

### 3.1 Baseline characteristics

**Table 1** presents the characteristics of the 4,977 participants, with a mean age of 40.2 ± 11.6 years, of whom 53.3% were female. Among the study population, 398 participants had gallstones, resulting in a prevalence rate of 8.0%. The mean ± SD of CBC-derived inflammatory biomarkers (SIRI, SII, NLR, PLR, MLR, and PIV) were 1.2 ± 0.9, 512.4 ± 314.6, 2.0 ± 1.1, 121.8 ± 48.2, 0.3 ± 0.1, and 301.8 ± 261.2, respectively. Additionally, BMI, SIRI, SII, NLR, CRP, and PIV levels were significantly higher in participants with gallstones than in those without.

**Table 1:** Baseline characteristics of study participants.

Characteristics	Total (N=4977)	Without Gallstones (N=4579)	With Gallstones (N=398)	P Value
**Age (year), mean ± SD**	40.2 ± 11.6	39.8 ± 11.6	44.3 ± 10.0	<0.0001
**Gender, n (%)**				<0.0001
Male	2323 (46.7)	2238 (48.9)	85 (21.4)	
Female	2654 (53.3)	2341 (51.1)	313 (78.6)	
**Race, n (%)**				0.0212
Mexican American	700 (14.1)	638 (13.9)	62 (15.6)	
Other Hispanic	535 (10.7)	487 (10.6)	48 (12.1)	
Non-Hispanic White	1512 (30.4)	1371 (29.9)	141 (35.4)	
Non-Hispanic Black	1265 (25.4)	1177 (25.7)	88 (22.1)	
Other Race	965 (19.4)	906 (19.8)	59 (14.8)	
**Education level, n (%)**				0.4218
Less than high school	831 (16.7)	762 (16.6)	69 (17.3)	
High school	1158 (23.3)	1076 (23.5)	82 (20.6)	
More than high school	2987 (60.0)	2740 (59.9)	247 (62.1)	
**Marital status, n (%)**				0.2030
Cohabitation	2951 (59.3)	2703 (59.0)	248 (62.3)	
Solitude	2025 (40.7)	1875 (41.0)	150 (37.7)	
**PIR, mean ± SD**	2.6 ± 1.7	2.6 ± 1.7	2.4 ± 1.6	0.0298
**Smoking status, n (%)**				0.0058
Yes	1873 (37.6)	1698 (37.1)	175 (44.1)	
No	3102 (62.4)	2880 (62.9)	222 (55.9)	
**Drinking status, n (%)**				<0.0001
Yes	2405 (56.2)	2255 (57.3)	150 (43.2)	
No	1877 (43.8)	1680 (42.7)	197 (56.8)	
**Diabetes, n (%)**				<0.0001
Yes	431 (8.7)	361 (7.9)	70 (17.6)	
No	4543 (91.3)	4215 (92.1)	328 (82.4)	
**Hypertension, n (%)**				<0.0001
Yes	1273 (25.6)	1116 (24.4)	157 (39.4)	
No	3699 (74.4)	3458 (75.6)	241 (60.6)	
**BMI (kg/m^2^), mean ± SD**	30.2 ± 7.9	29.8 ± 7.6	35.3 ± 9.9	<0.0001
**SIRI, mean ± SD**	1.2 ± 0.9	1.2 ± 0.9	1.3 ± 0.9	0.0185
**SII, mean ± SD**	512.4 ± 314.6	505.2 ± 305.7	596.1 ± 393.6	<0.0001
**NLR, mean ± SD**	2.0 ± 1.1	2.0 ± 1.1	2.2 ± 1.3	0.0009
**PLR, mean ± SD**	121.8 ± 48.2	121.3 ± 46.8	127.1 ± 62.5	0.2041
**MLR, mean ± SD**	0.3 ± 0.1	0.3 ± 0.1	0.3 ± 0.2	0.2290
**CRP, mean ± SD**	4.1 ± 8.4	3.9 ± 8.1	6.7 ± 10.7	<0.0001
**PIV, mean ± SD**	301.8 ± 261.2	297.4 ± 256.5	352.5 ± 305.3	<0.0001

*PIR, poverty income ratio; BMI, body mass index; SIRI, systemic inflammation response index; SII, systemic immune-inflammation index; NLR, neutrophil-to-lymphocyte ratio; MLR, monocyte-to-lymphocyte ratio; PLR, platelet-to-lymphocyte ratio; PIV, pan immune inflammatory value; CRP, C-reactive protein.

### 3.2 Association between CBC-derived inflammatory biomarkers and gallstones

**Table 2** shows the results of multivariate regression analysis for CBC-derived inflammatory biomarkers and gallstones risk. In models 1 and 2, all inflammatory biomarkers except for PLR and MLR demonstrated a positive correlation with the risk of gallstones. After adjusting for all included confounding factors, individuals in the highest quartiles of SIRI, SII, MLR, and PIV exhibited a higher risk of gallstones compared to those in the lowest quartiles. Individuals in the highest quartiles (Q4) of SIRI, SII, MLR, and PIV exhibited an increased risk of gallstones by 65% [OR (95%CI): 1.65(1.12,2.43)], 53% [OR (95%CI): 1.53(1.05,2.25)], 66% [OR (95%CI): 1.66(1.16,2.37)], and 82% [OR (95%CI): 1.82(1.23,2.71)], respectively, compared to those in the lowest quartiles (Q1).

**Table 2:** Association between CBC-derived inflammatory biomarkers and gallstones.

Exposure	Model 1 OR(95%CI)	Model 2 OR(95%CI)	Model 3 OR(95%CI)
SIRI (quartile)			
Q1	1.00(Reference)	1.00(Reference)	1.00(Reference)
Q2	1.19(0.87,1.63)	1.23(0.89,1.69)	1.29(0.87,1.92)
Q3	1.37(1.01,1.86)	1.47(1.08,2.02)	1.62(1.10,2.38)
Q4	1.64(1.22,2.20)	1.80(1.33,2.46)	1.65(1.12,2.43)
SII (quartile)			
Q1	1.00(Reference)	1.00(Reference)	1.00(Reference)
Q2	1.31(0.95,1.8)	1.27(0.91,1.76)	1.34(0.90,1.99)
Q3	1.37(1.00,1.88)	1.24(0.89,1.72)	1.14(0.76,1.69)
Q4	2.11(1.57,2.84)	1.79(1.31,2.44)	1.53(1.05,2.25)
NLR (quartile)			
Q1	1.00(Reference)	1.00(Reference)	1.00(Reference)
Q2	1.10(0.81,1.49)	1.05(0.76,1.44)	1.08(0.74,1.58)
Q3	1.10(0.81,1.49)	1.06(0.77,1.45)	1.02(0.69,1.48)
Q4	1.51(1.13,2.01)	1.40(1.04,1.89)	1.30(0.90,1.87)
PLR (quartile)			
Q1	1.00(Reference)	1.00(Reference)	1.00(Reference)
Q2	0.86(0.64,1.16)	0.83(0.61,1.13)	0.91(0.63,1.31)
Q3	0.89(0.66,1.20)	0.80(0.59,1.08)	0.82(0.57,1.18)
Q4	1.19(0.90,1.57)	0.97(0.73,1.29)	1.27(0.91,1.78)
MLR (quartile)			
Q1	1.00(Reference)	1.00(Reference)	1.00(Reference)
Q2	0.92(0.69,1.22)	0.98(0.73,1.31)	1.05(0.73,1.51)
Q3	0.78(0.58,1.05)	0.91(0.67,1.23)	1.22(0.85,1.76)
Q4	0.96(0.72,1.27)	1.19(0.88,1.61)	1.66(1.16,2.37)
CRP (quartile)			
Q1	1.00(Reference)	1.00(Reference)	1.00(Reference)
Q2	1.96(1.37,2.80)	1.79(1.24,2.57)	1.44(0.94,2.21)
Q3	1.96(1.37,2.80)	1.65(1.14,2.38)	0.99(0.63,1.54)
Q4	3.87(2.78,5.39)	2.93(2.09,4.13)	1.28(0.82,2.01)
PIV (quartile)			
Q1	1.00(Reference)	1.00(Reference)	1.00(Reference)
Q2	1.65(1.19,2.27)	1.71(1.23,2.38)	1.80(1.20,2.70)
Q3	1.58(1.14,2.18)	1.55(1.11,2.17)	1.51(1.00,2.27)
Q4	2.22(1.63,3.03)	2.21(1.61,3.04)	1.82(1.23,2.71)

Model 1: no covariates were adjusted;

Model 2: age, gender, and race were adjusted;

Model 3: age, gender, race, BMI, education level, marital status, PIR, diabetes, hypertension, smoking and drinking status were adjusted.

**Figure 2:**
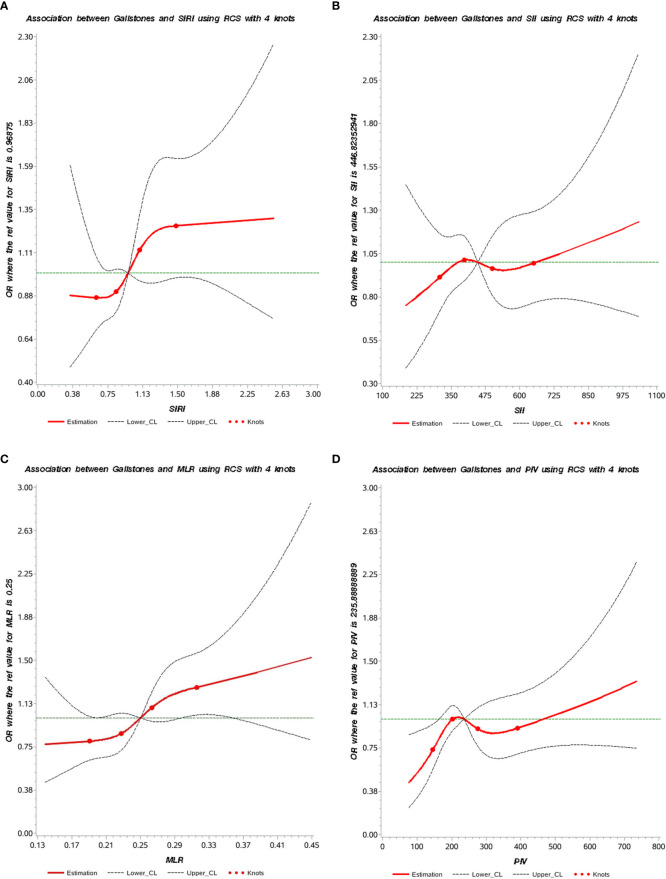
The nonlinear associations between SIRI, SII, MLR, PIV, and gallstones. **(A)** Association between SIRI and gallstones; **(B)** Association between SII and gallstones; **(C)** Association between MLR and gallstones; **(D)** Association between PIV and gallstones. The red solid lines represent the smooth curve fit between the variables, while the black dashed lines indicate the fitted 95% confidence intervals.

The RCS plots illustrated the nonlinear relationships between SIRI, SII, MLR, PIV, and the risk of gallstones (**Figure 2**).

### 3.3 Subgroup analysis

**Figure 3:**
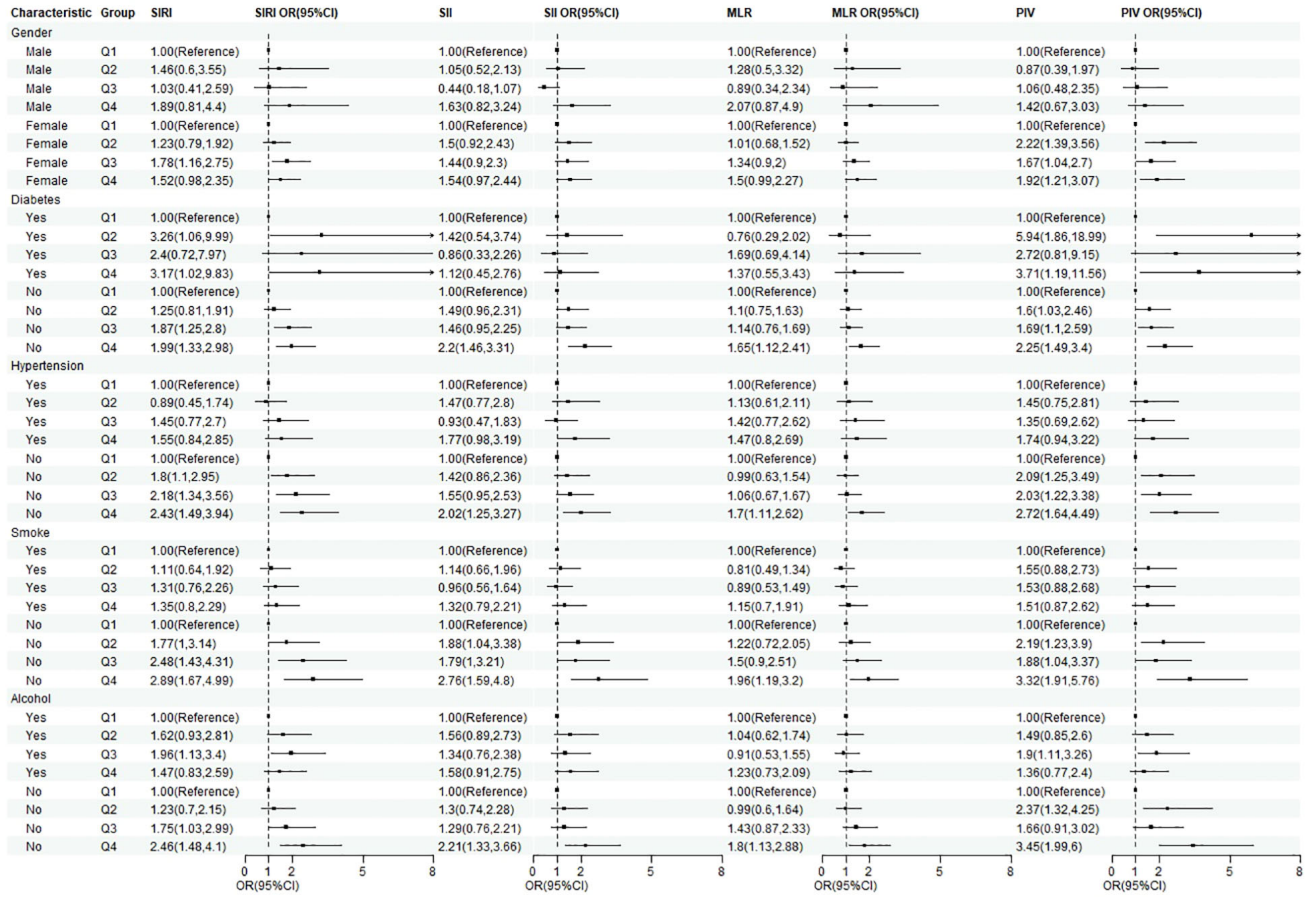
Subgroup analysis of the relationship between SIRI, SII, MLR, PIV, and gallstones.

To further assess the consistency of the associations between SIRI, SII, MLR, PIV, and gallstones across different populations, we conducted subgroup analyses (**Figure 3**). In most subgroups, the associations between various inflammatory biomarkers and gallstones remained robust.

## 4 Discussion

This study comprehensively investigated the relationship between CBC-derived inflammatory biomarkers and gallstones. The findings confirmed a nonlinear positive correlation between SIRI, SII, MLR, PIV, and the risk of gallstones. Subgroup analyses further established the robustness of these associations across different populations. Importantly, given their simplicity and accessibility in clinical practice, these inflammatory biomarkers hold significant clinical potential.

Previous studies have demonstrated an association between gallstone formation and inflammation (9, 19). The mechanism by which inflammation promotes gallstone formation likely involves changes in protein and lipid metabolism, which impact cholesterol and bile acid metabolism, ultimately leading to elevated bile salt levels conducive to gallstone development. In one of the largest studies on cytokines and gallstones, Liu et al. identified four circulating interleukins (IL-6, IL-10, IL-12, IL-13) associated with gallstones. It has also been shown the formation of gallstones can be promoted in mice by the administration of pro-inflammatory cytokines (IL-1). A study of 95,319 participants further demonstrated that elevated hs-CRP levels were an independent risk factor for incident gallstones in a Chinese population (20). In addition, a cohort study of 2,650 participants found significant associations between gallstones and both CRP levels and white blood cell count (11). Riveras et al. identified elevated transcript levels of genes involved in inflammatory and immune pathways among patients with gallstones (21). The dietary inflammatory index (DII) assesses the inflammatory potential of an individual’s diet based on levels of inflammatory cytokines in the blood (22), and studies indicate that pro-inflammatory diets may promote gallstone development (23, 24). Most prior studies have focused on risk factors for gallstones in individuals over 60 years old. However, as gallstone incidence rises in younger populations, this study specifically examined the relationship between inflammatory biomarkers and gallstones in adults under 60. By identifying the role of inflammatory markers in this age group, we provide new insights into early risk stratification and potential prevention strategies.

As indicators derived from routine laboratory evaluations, CBC-derived inflammatory biomarkers encompass blood components such as lymphocytes, monocytes, neutrophils, and platelets, offering a comprehensive view of the immune-inflammatory state. Neutrophils, being key components of the innate immune system, play a pivotal role in pathogen elimination through the widespread release of neutrophil extracellular traps (NETs) (25). The formation of NETs can activate the innate immune system and promote the development and growth of gallstones (26). Monocytes contribute to immune defense and tissue repair, while lymphocytes regulate the immune system by secreting cytokines and cytolytic activity. Inflammatory cytokines can alter the absorptive and secretory functions of gallbladder epithelial cells, subsequently increasing the risk of gallstone formation (27). In addition, inflammatory factors such as TNF-α can promote the expression of mucins, contributing to gallstone formation (28, 29). A review by Maurer et al. summarized relevant studies on the immune system’s potential role in gallstone formation, suggesting that inflammatory mediators or their subsets could serve as biomarkers for gallstones (9).

This study is the first pioneering research to focus on the relationship between CBC-derived inflammatory biomarkers and gallstones in U.S. adults under 60 years old. Adjustments for confounding variables and subgroup analyses enhance the reliability of the findings. Early identification of individuals at risk for gallstones through easily obtainable inflammatory biomarkers, such as SIRI, SII, MLR, and PIV, could facilitate timely interventions, including lifestyle modifications and closer monitoring. However, this study has several limitations. First, its cross-sectional design precludes establishing a causal relationship between CBC-derived inflammatory biomarkers and gallstones. Second, despite comprehensive adjustment for multiple covariates, unmeasured confounding factors may still influence the results. Third, gallstone identification relied on participants’ self-reported responses to a specific question, potentially introducing recall and interviewer bias. Fourth, as this study focuses on American adults under the age of 60, further research is needed to validate the applicability of these findings to other populations. Lastly, the use of single-time CBC parameters to calculate CBC-derived inflammatory biomarkers may introduce potential bias.

## 5 Conclusions

Our findings indicate that for adults under 60 years old in the United States, SIRI, SII, MLR, and PIV can serve as predictive indicators for the occurrence of gallstones.

## Data Availability

Publicly available datasets were analyzed in this study. This data can be found here: https://www.cdc.gov/nchs/nhanes.
